# Cumulative surgeon volume is associated with improved outcomes following hip arthroscopy: A national registry study

**DOI:** 10.1002/ksa.70573

**Published:** 2026-08-27

**Authors:** Richard Holleyman, William Cawley, Tim Board, Marcus Bankes, Antonio Andrade, Andrew Judge, Vikas Khanduja, Bernard van Duren, Ajay Malviya

**Affiliations:** ^1^ Newcastle University Newcastle Upon Tyne UK; ^2^ Wrightington Hospital Wigan & Leigh NHS Foundation Trust Wigan UK; ^3^ Fortius Clinic London UK; ^4^ Royal Berkshire Hospital Royal Berkshire NHS Foundation Trust Berkshire UK; ^5^ Bristol University Bristol UK; ^6^ Addenbrookes Hospital Cambridge University Hospitals NHS Foundation Trust Cambridge UK; ^7^ Nottingham University Hospitals NHS Trust, Trust Headquarters City Hospital Campus Nottingham Nottinghamshire UK; ^8^ Northumbria Healthcare NHS Foundation Trust Newcastle upon Tyne UK

**Keywords:** femoroacetabular impingement, hip arthroscopy, outcomes, PROMs, surgeon volume

## Abstract

**Purpose:**

This study assessed the association between (1) annual and (2) cumulative surgeon volume and patient‐reported outcome measures (PROMS) after hip arthroscopic surgery for femoroacetabular impingement syndrome (FAIS) using data from the UK Nonarthroplasty hip registry (NAHR).

**Methods:**

All hip arthroscopies (HAs) recorded in the UK NAHR between January 2012 and May 2023 were identified. Annual and cumulative consultant HA volume were derived for each case. International Hip Outcome Tool (iHOT)‐12 and EuroQol‐5 Dimensions (EQ‐5D) index scores were captured preoperatively and at 6 and 12 months postoperatively. Multivariable linear regression models with restricted cubic splines were used to define associations between consultant annual and cumulative volume and outcome. The null hypothesis was that neither annual nor cumulative volume would be significantly associated with postoperative iHOT‐12 or EQ‐5D score change.

**Results:**

A total of 3531 HAs were identified (median age 36 years [IQR: 28–44], 62.4% female) from 76 contributing consultant surgeons. Median annual consultant HA volume was 51 (IQR: 22–102), and median cumulative volume was 163 (IQR: 57–404). Cumulative consultant volume of 170 HA cases demonstrated the lowest 12‐month postoperative iHOT‐12 score gain (29.6, confidence interval [CI: 27.0–32.1]) compared to a cumulative consultant HA volume of 972 cases (35.2 [95%CI 32.1–38.3]), where the score difference became significant but remained below the minimal clinically important difference (MCID). No statistically significant relationship between consultant volume and EQ‐5D Index were observed. Cases where the consultant had the highest cumulative volumes were younger (35.7 vs. 37.0 years, *p* = 0.03) and a greater proportion underwent excision of isolated cam impingement lesions (62.2% vs. 49.3% *p* = 0.001), compared to cases performed by the lowest volume consultants.

**Conclusion:**

This study found a small but statistically significant association (below the MCID) between higher cumulative case volume and improved early functional outcome following HA. Highest‐volume surgeons consistently treated younger patients with less‐severe chondral damage.

**Level of Evidence:**

Level III.

**Abbreviations**
EQ‐5D: EuroQol‐5 dimensions
FAI: femoroacetabular impingement
HA: hip arthroscopy
iHOT‐12: 12‐question international hip outcome tool
iHOT‐33: international hip outcome tool
IQR: interquartile range
MCID: minimally‐important clinical difference
NAHR: nonarthroplasty hip registry
PROM(s): patient reported outcome measure(s)
SCB: substantial clinical benefit
THA: total hip arthroplasty

## INTRODUCTION

Hip arthroscopy (HA) has become increasingly utilised in the management of femoroacetabular impingement syndrome (FAIS) [3, 14, 19, 34, 44, 45], as well as to manage septic arthritis and other paediatric and adolescent articular and periarticular hip problems [19]. It is an attractive option due to its minimally invasive nature and reduced recovery times while maintaining acceptable outcomes, especially in younger patients and athletes [19, 53]. The number of hip arthroscopies being performed and the reported literature related to HA is continuing to grow [17]. HA is a technically challenging procedure, with a steep learning curve. Factors such as high level of joint congruency, complexity of surrounding tissues, the depth of the hip joint and the risks of distraction during surgery all contribute to the technical difficulty of the procedure [14, 17, 19, 26, 48]. While generally reported to produce low complication rates [3, 45], HA has also been associated with rare, but major surgical complications such as avascular necrosis of the femoral head, fracture of the femoral neck and postoperative hip dislocation [19, 45, 52].

Existing studies investigating the effects of surgeon volume on outcomes after HA are limited by small patient cohorts, small numbers of surgeons and single centres reporting data, and as a result their findings lack generalisability and reliability. Much of the existing literature is focussed around the learning curve, assessing improvements in competency using a ‘split group method’, where after a certain cumulative case volume threshold, a surgeon becomes ‘proficient’ in the procedure and outcomes after this point are more favourable than outcomes before this point [50]. However, the learning curve threshold for proficiency for HA remains arbitrary and poorly defined in the literature [17, 48], with studies reporting the case threshold to achieve improved outcomes ranging from 30 to 500 cases [17, 20, 26, 35, 46, 48, 53]. Therefore, studies assessing outcomes before and after these cut‐offs are limited by the definition of each study of the case volume required to achieve competency. Understanding the relationship between surgeon volume and outcomes has direct clinical relevance, with implications for service configuration, surgical credentialing and patient referral pathways. The present study addresses the limitations of previous work by using national registry data from the largest cohort of surgeons and patients reported to date and by modelling volume as a continuous exposure, thereby avoiding arbitrary case‐number thresholds and allowing assessment across the full continuum of surgeon experience.

Therefore, the purpose of this study was to assess the association between (1) annual and (2) cumulative surgeon volume and patient‐reported outcome measures (PROMs) after hip arthroscopic surgery for FAIS using data from the UK NonArthroplasty Hip Registry (NAHR). The null hypothesis was that there was no relationship between either surgeon experience metric and PROMS.

## METHODS

Ethical approval for this cohort study was obtained prior to initiation. We received and included data for all hip arthroscopies recorded in the NAHR between 1 January 2012 and 31 April 2023. Informed consent for inclusion was obtained from all patients recorded in the NAHR, including for using their data in service evaluation and research.

Cases were included for patients aged 16 years or older undergoing HA for FAIS. In the experience of the authors, patients with radiographic acetabular dysplasia (generally defined as a lateral centre‐edge angle <25 degrees) and those with inflammatory or rheumatological arthropathy are not routinely offered HA for FAIS in UK practice and were therefore unlikely to be represented in this cohort. Additional exclusion criteria included previous surgery to the ipsilateral hip, concomitant non‐FAIS procedures in the index procedure, unclassified procedure details, erroneous pathway chronology, no details on sex and no availability of the preoperative PROMs questionnaire and at least one linked postoperative questionnaire. The NAHR does not routinely record Tönnis osteoarthritis grade, formal dysplasia parameters, concurrent knee osteoarthritis or previous trauma, and these variables were therefore not available for analysis.

Demographic, diagnostic and procedural data are collected using a structured online form when recorded in the NAHR. FAIS type was categorised based upon the index procedure performed for FAIS and included (1) ‘cam’—excision of cam lesion (with no recorded procedure to treat pincer pathology during the same surgery), (2) ‘pincer’—excision pincer lesion (with no recorded procedure to treat cam pathology during the same surgery), (3) ‘mixed’—excision of both a cam and pincer lesion during the same operation and (4) ‘none’—no impingement lesion treated during the index HA. Where recorded, acetabular chondral damage was determined as the single worst area of cartilage damage recorded intraoperatively according to Konan et al.'s description (Grade 1—wave sign with intact chondrolabral junction, Grade 2—Chondrolabral junction separation but no delamination, Grade 3—Delamination, Grade 4—Exposed bone) [18, 24]. Femoral cartilage defects were classified according to the Outerbridge classification [34].

### Outcomes

The NAHR invites patients to complete online questionnaires to capture PROMs, including the EuroQol‐5 dimensions (EQ‐5D) Index and the 12‐question International Hip Outcome Tool (iHOT)‐12, preoperatively, at 6 and 12 months following HA. The iHOT‐12 score is an abbreviated, validated version of the iHOT‐33, which can be utilised in the assessment of nonarthritic hip problems in young, active patients [11, 42]. The iHOT‐12 consists of 12 questions related to four key domains of daily life (symptoms and functional limitations, sport and recreational activities, job‐related concerns and social, emotional and lifestyle concerns). The EQ‐5D Index is a generic measure of health status developed to assess clinical and economic evaluations of healthcare [37]. The EQ‐5D consists of self‐classifier statements from five domains (mobility, self‐care, usual activities, pain/discomfort and anxiety/depression) and a visual analogue scale (0 = worst imaginable health state, 100 = best imaginable health state) describing the patient's view of their overall health.

For patients in whom pre and postoperative outcome scores were recorded at 12‐month follow‐up, improvements in iHOT‐12 score of ≥13 and ≥28 were used as reference criteria for achieving minimum clinically important difference (MCID) and substantial clinical benefit (SCB), respectively as reported by Martin et al. and Kivlan et al. [23, 29]. The primary outcome was the change in iHOT‐12 score from baseline to 12 months postoperatively. Secondary outcomes were the change in EQ‐5D Index score and the proportions of patients achieving the MCID and SCB for the iHOT‐12.

### Main exposure

Our main exposures were: (1) annual, and (2) cumulative volume of HA undertaken by the consultant (i.e., surgeon who has completed training) surgeon at the time of the index HA procedure. We utilised continuous data analysis to eliminate the issues of arbitrary case number cut‐offs and to allow for assessment of outcomes across the continuum of surgeon experience, mirroring more closely the process of constant improvement and technique refinement anecdotally experienced by many surgeons. Annual or cumulative volume to date was derived individually for each case, capturing changes in consultant surgeon volume that vary with time. Annual and cumulative volumes in totality for all HA indications (and prior to the study cohort exclusions—Figure 1) were derived. Thus, for each HA in the eligible study population, two metrics were determined:

‐ Consultant surgeon annual volume of HA—the number of HA cases (for any indication) recorded by the responsible consultant in NAHR in the 365 days before the index case.
‐ Consultant surgeon cumulative volume of HA—the total cumulative volume of HA recorded by the responsible consultant in the NAHR before the day the index case was undertaken, and since 1st January 2012.

**Figure 1:**
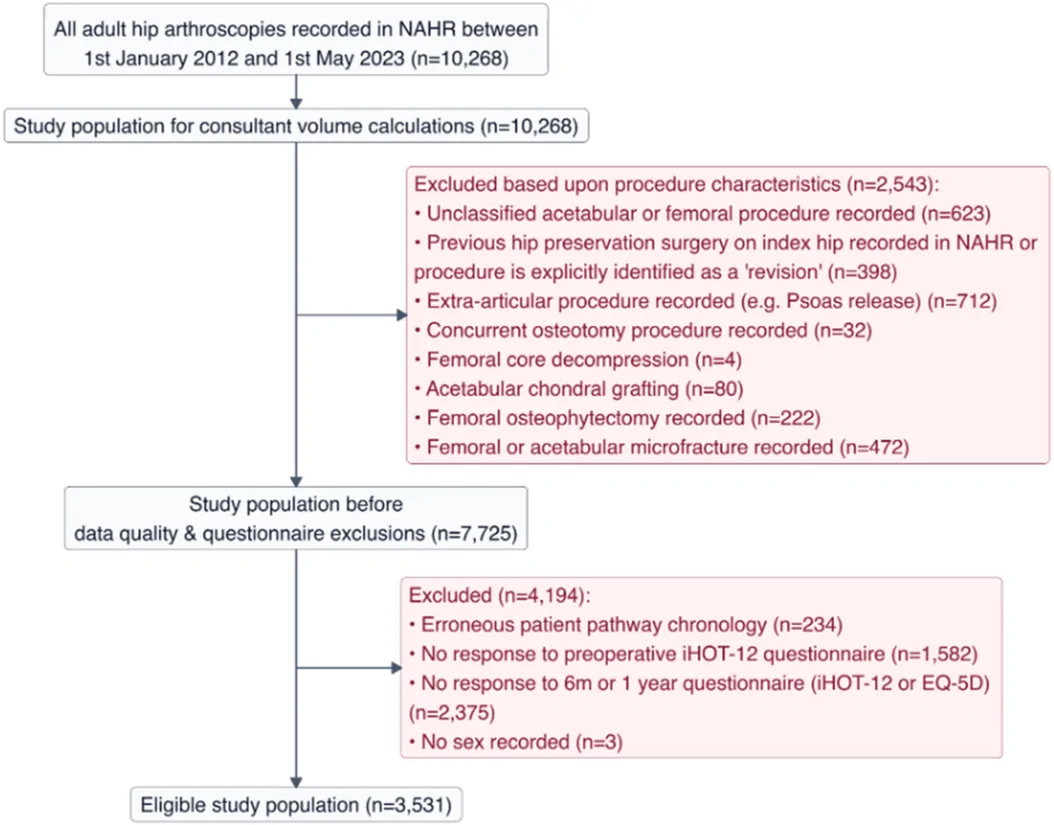
Study flow diagram. EQ‐5D, EuroQol‐5 dimensions index; iHOT‐12, 12‐question international hip outcome tool; NAHR, nonarthroplasty Hip Registry.

### Statistical analysis

To explore how case‐mix characteristics and outcomes varied with annual and cumulative consultant volume, cases in the study cohort were grouped into quartiles of annual and cumulative consultant surgeon HA volume. Between‐group comparisons were made using the Chi‐squared test for categorical data and Kruskal–Wallis (nonparametric data) or analysis of variance (parametric data) tests for between‐group comparisons of continuous data. Pairwise comparisons of categorical data between quartile groups were made using the Chi‐squared test with a Bonferroni correction for multiple comparisons. Pairwise comparisons of continuous data were made using the student's t test or the Wilcoxon test for normally and nonnormally distributed data, respectively. Within‐group comparisons of paired continuous data (pre vs. postoperative score change [delta]) were derived using the paired t‐test.

Multivariable generalised linear models were used to investigate the relationship between HA volume and iHOT‐12 and EQ‐5D Index score change at 6 and 12 months, adjusting for age, sex, FAIS type, preoperative iHOT‐12 score and acetabular chondral injury grade. Volume was modelled as a continuous variable using restricted cubic splines to explore and characterise any nonlinear relationships or threshold effects. Knots were prespecified using the 5th, 27.5th, 50th, 72.5th and 95th centiles of the volume distribution. Analyses were performed in STATA (Stata Statistical Software: Release 15) and R, version 4 (Vienna, Austria). The quartile‐based and spline‐based analyses were considered complementary rather than contradictory: categorising a continuous exposure into quartiles reduces statistical power and introduces within‐group heterogeneity, whereas modelling volume continuously with restricted cubic splines is more statistically efficient and better able to detect nonlinear trends and threshold effects that quartile grouping may obscure. A formal a priori power calculation was not performed, as is conventional for registry‐based observational cohort studies in which the sample size is determined by the available data rather than by experimental design; the large sample (*n* = 3531) provided substantial power to detect clinically relevant associations.

## RESULTS

A total of 3531 cases of HA identified from the NAHR database after exclusions (Figure 1). Median patient age was 36 years (IQR: 28–44), and 62.4% (2202/3531) of patients were female. Seventy‐six unique consultant surgeons contributed cases,with a median annual volume of HA of 51 (IQR: 22–102) and a median cumulative volume of 163 (IQR: 57–404). Case characteristics are summarised by annual and cumulative consultant volume quartiles in Tables 1 and 2, respectively. Postoperative iHOT‐12 scores were available for 76.3% (2695/3531) of cases in the study cohort at 6 months and 71.4% (2520/3531) at 12 months follow‐up.

**Table 1:** Case characteristics by annual consultant volume of the responsible consultant at the time the arthroscopy was performed.

	Annual consultant volume group [*n* (%)]				
	0–22	23–51	52–102	103–197	
Variable	*n* = 889 (25.2)	*n* = 891 (25.2)	*n* = 889 (25.2)	*n* = 862 (24.4)	*p*‐Value
Annual arthroscopy volume [median (IQR)]	11 (5, 17)	36 (29, 42)	72 (64, 83)	131 (113, 161)	<0.0001
Cumulative arthroscopy volume [median (IQR)]	27 (11, 73)	90 (52, 176)	215 (132, 461)	503 (313, 851)	<0.0001
Number of unique consultants	75	35	16	6	
Age in years test [mean (SD)]	36.8 (10.4)	35.7 (10.3)	35.9 (10.4)	36.0 (10.6)	0.10
Sex [*n* (%)]					0.85
Female	544 (61.2)	563 (63.2)	557 (62.7)	538 (62.4)	
Male	345 (38.8)	328 (36.8)	332 (37.3)	324 (37.6)	
BMI [mean (SD); *n* (%) with available data] (kg/m^2^)	25.7 (4.6); *n *= 488 (55)	26.1 (5.1); *n *= 564 (63)	26.0 (4.6); *n *= 714 (80)	25.1 (4.4); *n *= 671 (78)	<0.0001
FAIS type [*n* (%)]					<0.0001
Cam	464 (52.2)	375 (42.1)	521 (58.6)	510 (59.2)	
Pincer	57 (6.4)	79 (8.9)	42 (4.7)	39 (4.5)	
Mixed	204 (22.9)	279 (31.3)	227 (25.5)	194 (22.5)	
None	164 (18.4)	158 (17.7)	99 (11.1)	119 (13.8)	
Severity of single worst zone of acetabular chondral damage [*n* (%)]					0.00085
No chondral damage	145 (21.1)	74 (11.5)	119 (16.8)	119 (18.2)	
Grade 1—Wave Sign with intact chondrolabral junction	149 (21.7)	167 (26.0)	140 (19.7)	151 (23.1)	
Grade 2—Chondrolabral junction separation but no delamination	164 (23.9)	159 (24.8)	175 (24.6)	163 (24.9)	
Grade 3—Cartilage Delamination	173 (25.2)	193 (30.1)	221 (31.1)	164 (25.0)	
Grade 4—Exposed bone	56 (8.2)	49 (7.6)	55 (7.7)	58 (8.9)	
Not recorded	202	249	179	207	
Femoral chondral damage grade (Outerbridge) [*n* (%)]					<0.0001
No chondral damage	369 (57.7)	373 (65.0)	495 (84.0)	477 (73.0)	
Grade 1—Rough surface, chondral softening	164 (25.6)	125 (21.8)	18 (3.1)	37 (5.7)	
Grade 2—Irregular Surface defects <50% Cartilage Depth	68 (10.6)	44 (7.7)	43 (7.3)	73 (11.2)	
Grade 3—>50% loss of cartilage depth	30 (4.7)	23 (4.0)	22 (3.7)	38 (5.8)	
Grade 4—full‐thickness loss	9 (1.4)	9 (1.6)	11 (1.9)	28 (4.3)	
Not recorded	249	317	300	209	

*Note*: Cases were binned into quartiles of annual volume. Cases with missing chondral grading data were excluded from column percentage calculations. *n* (%) indicates the number of nonmissing data points for BMI by group.

Abbreviations: BMI, body mass index; FAIS, femoroacetabular impingement; IQR, interquartile range; SD, standard deviation.

**Table 2:** Case characteristics by cumulative consultant volume of the responsible consultant at the time the arthroscopy was performed.

	Cumulative consultant volume group [*n* (%)]				
Variable	0–57 *n* = 885 (25.1)	58–163 *n* = 885 (25.1)	164–404 *n* = 879 (24.9)	405–1520 *n* = 882 (25.0)	*p*‐Value
Annual arthroscopy volume [median (IQR)]	14 (6, 26)	43 (29, 64)	77 (41, 117)	109 (76, 147)	<0.0001
Cumulative arthroscopy volume [median (IQR)]	25 (11, 40)	102 (77, 131)	254 (199, 322)	669 (518, 1054)	<0.0001
Number of unique consultants	71	37	20	5	
Age in years test [mean (SD)]	37.0 (10.3)	35.7 (10.5)	36.0 (10.4)	35.7 (10.6)	0.033
Sex [*n* (%)]					0.13
Female	579 (65.4)	532 (60.1)	544 (61.9)	547 (62.0)	
Male	306 (34.6)	353 (39.9)	335 (38.1)	335 (38.0)	
BMI [mean (SD); *n* (%) with available data] (kg/m^2^)	25.6 (4.7); *n* = 415 (47)	26.0 (4.8); *n* = 568 (64)	26.2 (5.0); *n* = 666 (76)	25.1 (4.3); *n* = 788 (89)	<0.0001
FAIS type [*n* (%)]					<0.0001
Cam	436 (49.3)	400 (45.2)	485 (55.2)	549 (62.2)	
Pincer	68 (7.7)	73 (8.2)	34 (3.9)	42 (4.8)	
Mixed	222 (25.1)	261 (29.5)	239 (27.2)	182 (20.6)	
None	159 (18.0)	151 (17.1)	121 (13.8)	109 (12.4)	
Severity of single worst zone of acetabular chondral damage^a^ [*n* (%)]					<0.0001
No chondral damage	130 (22.5)	56 (8.7)	101 (14.0)	170 (22.8)	
Grade 1—Wave sign with intact chondrolabral junction	119 (20.6)	172 (26.6)	172 (23.8)	144 (19.3)	
Grade 2—Chondrolabral junction separation but no delamination	143 (24.8)	184 (28.4)	189 (26.1)	145 (19.4)	
Grade 3—Cartilage delamination	137 (23.7)	192 (29.7)	197 (27.2)	225 (30.1)	
Grade 4—Exposed bone	48 (8.3)	43 (6.6)	64 (8.9)	63 (8.4)	
Not recorded	308	238	156	135	
Femoral chondral damage grade (Outerbridge) [*n* (%)]					<0.0001
No chondral damage	292 (55.1)	403 (68.3)	440 (70.9)	579 (81.0)	
Grade 1—Rough surface, chondral softening	144 (27.2)	101 (17.1)	71 (11.4)	28 (3.9)	
Grade 2—Irregular Surface defects <50% cartilage depth	55 (10.4)	63 (10.7)	66 (10.6)	44 (6.2)	
Grade 3—>50% loss of cartilage depth	28 (5.3)	20 (3.4)	30 (4.8)	35 (4.9)	
Grade 4—Full‐thickness loss	11 (2.1)	3 (0.51)	14 (2.3)	29 (4.1)	
Not recorded	355	295	258	167	

*Note*: Cases were binned into quartiles of cumulative volume. Cases with missing chondral grading data were excluded from column percentage calculations. *n* (%) indicates the number of nonmissing data points for BMI by group.

Abbreviations: BMI, body mass index; FAIS, femoroacetabular impingement; IQR, interquartile range; SD, standard deviation.

a Chondral damage graded according to Konan et al. (see methods).

Patients in the highest cumulative volume group were significantly younger, and a greater proportion underwent excision of an isolated cam impingement lesion compared to cases in the lowest volume group, where a greater proportion of cases were recorded as pincer, mixed and non‐FAIS lesions. Additionally, a greater proportion of cases in the highest cumulative volume group demonstrated no chondral damage at time of procedure (*p* < 0.0001).

### Univariate analysis

Preoperative iHOT‐12 scores were significantly higher in the highest volume quartile group compared to the lowest volume quartile for both annual (*p* < 0.001) and cumulative volume (*p* < 0.001) (Tables 3 and 4). Postoperative iHOT‐12 scores at 12 months were significantly higher in the highest annual volume quartile group compared to second lowest quartile (*p* = 0.031) (Table 3) and in the highest cumulative volume quartile group compared to the 1st and 2nd lowest cumulative volume quartiles (*p* < 0.001 and *p* = 0.006, respectively). There were no statistically significant differences in iHOT‐12 score improvement at 12 months between annual or cumulative volume quartiles.

**Table 3:** Outcomes by annual consultant surgeon volume of the responsible consultant at the time the arthroscopy was performed. Cases were binned into quartiles of annual volume.

	Annual consultant volume group [*n* (%)]			
Variable	0–22 *n* = 889 (25.2)	23–51 *n* = 891 (25.2)	52–102 *n* = 889 (25.2)	103–197 *n* = 862 (24.4)
Median outcome score, (IQR); *n* (%)				
Preop iHOT‐12	29.0 (18.0, 45.0); *n* = 889 (100.0%)	30.0 (19.0, 43.0); *n* = 891 (100.0%)	30.0 (19.0, 44.0); *n* = 889 (100.0%)	34.0 (22.0, 47.8); *n* = 862 (100.0%)
Preop EQ‐5D index	0.57 (0.34, 0.70); *n* = 859 (96.6%)	0.58 (0.35, 0.69); *n* = 876 (98.3%)	0.62 (0.36, 0.70); *n* = 875 (98.4%)	0.62 (0.42, 0.70); *n* = 853 (99.0%)
Preop EQ‐5D VAS	70.0 (50.0, 80.0); *n* = 870 (97.9%)	70.0 (51.0, 80.0); *n* = 877 (98.4%)	70.0 (50.0, 80.0); *n* = 877 (98.7%)	70.0 (58.0, 80.0); *n* = 853 (99.0%)
6‐month iHOT‐12	61.0 (35.0, 80.0); *n* = 686 (77.2%)	58.0 (35.0, 81.0); *n* = 639 (71.7%)	63.0 (39.0, 82.0); *n* = 697 (78.4%)	66.0 (41.0, 84.0); *n* = 673 (78.1%)
6‐month EQ‐5D index	0.71 (0.59, 0.80); *n* = 714 (80.3%)	0.71 (0.60, 0.80); *n* = 669 (75.1%)	0.73 (0.59, 0.80); *n* = 729 (82.0%)	0.74 (0.64, 0.84); *n* = 700 (81.2%)
6‐month EQ‐5D VAS	75.0 (60.0, 87.0); *n* = 717 (80.7%)	76.5 (60.0, 87.8); *n* = 670 (75.2%)	75.0 (60.0, 85.0); *n* = 729 (82.0%)	75.0 (64.0, 86.0); *n* = 700 (81.2%)
12‐month iHOT‐12	63.0 (33.0, 84.0); *n* = 648 (72.9%)	60.0 (33.0, 84.0); *n* = 651 (73.1%)	62.0 (38.0, 86.0); *n* = 609 (68.5%)	66.0 (39.0, 86.0); *n* = 612 (71.0%)
12‐month EQ‐5D index	0.73 (0.57, 0.80); *n* = 679 (76.4%)	0.71 (0.55, 0.84); *n* = 684 (76.8%)	0.74 (0.62, 0.84); *n* = 650 (73.1%)	0.74 (0.62, 0.84); *n* = 636 (73.8%)
12‐month EQ‐5D VAS	79.0 (59.0, 89.0); *n* = 684 (76.9%)	75.0 (59.0, 89.0); *n* = 683 (76.7%)	79.0 (60.0, 88.8); *n* = 650 (73.1%)	79.0 (64.0, 89.0); *n* = 636 (73.8%)
Mean change at 6‐month, (95%CI); *n* (%); *p* a				
iHOT‐12	+26.5 (24.6, 28.4); *n* = 686 (77.2%); <0.0001	+24.6 (22.6, 26.6); *n* = 639 (71.7%); <0.0001	+26.7 (24.8, 28.6); *n* = 697 (78.4%); <0.0001	+25.7 (23.7, 27.7); *n* = 673 (78.1%); <0.0001
EQ‐5D index	+0.16 (0.14, 0.18); *n* = 696 (78.3%); <0.0001	+0.15 (0.13, 0.17); *n* = 657 (73.7%); <0.0001	+0.15 (0.14, 0.17); *n* = 719 (80.9%); <0.0001	+0.14 (0.13, 0.16); *n* = 694 (80.5%); <0.0001
EQ‐5D VAS	+4.6 (2.8, 6.4); *n* = 700 (78.7%); <0.0001	+3.5 (1.7, 5.4); *n* = 659 (74.0%); 0.00024	+3.0 (1.2, 4.8); *n* = 721 (81.1%); 0.0010	+3.4 (1.5, 5.2); *n* = 694 (80.5%); 0.00043
% Achieving MCID iHOT‐12	67.2 (461 of 686)	64.2 (410 of 639)	70.0 (488 of 697)	68.1 (458 of 673)
% Achieving SCB iHOT‐12	45.0 (309 of 686)	45.9 (293 of 639)	47.2 (329 of 697)	48.0 (323 of 673)
Mean change at 12‐month, (95%CI); *n* (%); *p* a				
iHOT‐12	+24.8 (22.7, 26.9); *n* = 648 (72.9%); <0.0001	+25.6 (23.5, 27.8); *n* = 651 (73.1%); <0.0001	+26.9 (24.8, 29.0); *n* = 609 (68.5%); <0.0001	+26.7 (24.5, 28.8); *n* = 612 (71.0%); <0.0001
EQ‐5D index	+0.15 (0.13, 0.17); *n* = 664 (74.7%); <0.0001	+0.14 (0.12, 0.16); *n* = 673 (75.5%); <0.0001	+0.15 (0.13, 0.17); *n* = 642 (72.2%); <0.0001	+0.15 (0.13, 0.17); *n* = 630 (73.1%); <0.0001
EQ‐5D VAS	+4.3 (2.5, 6.2); *n* = 674 (75.8%); <0.0001	+2.0 (0.1, 4.0); *n* = 673 (75.5%); 0.044	+3.9 (2.0, 5.7); *n* = 643 (72.3%); <0.0001	+5.3 (3.3, 7.3); *n* = 630 (73.1%); <0.0001
% Achieving MCID iHOT‐12	63.4 (411 of 648)	64.1 (417 of 651)	68.6 (418 of 609)	67.8 (415 of 612)
% Achieving SCB iHOT‐12	46.0 (298 of 648)	47.5 (309 of 651)	47.3 (288 of 609)	48.5 (297 of 612)

Abbreviations: CI, confidence interval; EQ‐5D, EuroQol‐5 Dimensions (EQ‐5D) Index; iHOT‐12, 12‐question international hip outcome tool; IQR, interquartile range; MCID, minimum clinically important difference; Preop, preoperative; SCB, substantial clinical benefit; SD, standard deviation; VAS, visual analogue scale.

^a^The values are given as the mean score improvement (95% CI); number (%) of cases available for follow‐up; *p*‐value derived with the paired *t*‐test comparing pre versus postoperative score. MCID and SCB are predicated on pre and postoperative scores to be recorded.

**Table 4:** Outcomes by cumulative consultant volume of the responsible consultant at the time the arthroscopy was performed. Cases were binned into quartiles of cumulative volume.

	Cumulative consultant volume group [*n* (%)]			
Variable	0–57 *n* = 885 (25.1)	58–163 *n* = 885 (25.1)	164–404 *n* = 879 (24.9)	405–1520 *n* = 882 (25.0)
Median outcome score, (IQR); *n* (%)				
Preop iHOT‐12	27.0 (16.0, 41.0); *n* = 885 (100.0%)	30.0 (19.0, 43.0); *n* = 885 (100.0%)	31.0 (19.0, 46.0); *n* = 879 (100.0%)	34.0 (23.0, 48.0); *n* = 882 (100.0%)
Preop EQ‐5D index	0.56 (0.32, 0.69); *n* = 855 (96.6%)	0.58 (0.34, 0.70); *n* = 871 (98.4%)	0.62 (0.41, 0.71); *n* = 864 (98.3%)	0.62 (0.41, 0.70); *n* = 873 (99.0%)
Preop EQ‐5D VAS	70.0 (50.0, 80.0); *n* = 866 (97.9%)	70.0 (52.0, 80.0); *n* = 872 (98.5%)	70.0 (50.0, 80.0); *n* = 865 (98.4%)	70.0 (58.0, 80.0); *n* = 874 (99.1%)
6‐month iHOT‐12	58.0 (32.3, 78.0); *n* = 654 (73.9%)	61.0 (36.0, 84.0); *n* = 656 (74.1%)	66.0 (43.0, 85.0); *n* = 681 (77.5%)	64.0 (39.8, 81.0); *n* = 704 (79.8%)
6‐month EQ‐5D index	0.71 (0.56, 0.80); *n* = 685 (77.4%)	0.71 (0.60, 0.84); *n* = 678 (76.6%)	0.74 (0.64, 0.84); *n* = 715 (81.3%)	0.74 (0.62, 0.80); *n* = 734 (83.2%)
6‐month EQ‐5D VAS	75.0 (59.0, 86.0); *n* = 688 (77.7%)	76.0 (60.0, 88.0); *n* = 678 (76.6%)	78.0 (61.5, 88.0); *n* = 715 (81.3%)	75.0 (62.0, 85.0); *n* = 735 (83.3%)
12‐month iHOT‐12	60.0 (33.0, 83.0); *n* = 625 (70.6%)	60.0 (35.0, 84.0); *n* = 632 (71.4%)	63.0 (34.0, 86.0); *n* = 626 (71.2%)	66.0 (44.0, 86.0); *n* = 637 (72.2%)
12‐month EQ‐5D Index	0.71 (0.56, 0.80); *n* = 669 (75.6%)	0.72 (0.59, 0.84); *n* = 659 (74.5%)	0.73 (0.61, 0.84); *n* = 655 (74.5%)	0.74 (0.62, 0.84); *n* = 666 (75.5%)
12‐month EQ‐5D VAS	79.0 (57.0, 89.0); *n* = 674 (76.2%)	75.0 (58.0, 89.0); *n* = 659 (74.5%)	78.0 (63.0, 89.0); *n* = 655 (74.5%)	78.0 (64.0, 86.0); *n* = 665 (75.4%)
Mean change at 6‐month, (95%CI); *n* (%); *p* ^a^				
iHOT‐12	+25.8 (23.8, 27.7); *n* = 654 (73.9%); <0.0001	+26.2 (24.2, 28.2); *n* = 656 (74.1%); <0.0001	+27.8 (25.8, 29.8); *n* = 681 (77.5%); <0.0001	+23.9 (22.1, 25.7); *n* = 704 (79.8%); <0.0001
EQ‐5D Index	+0.16 (0.14, 0.18); *n* = 666 (75.3%); <0.0001	+0.16 (0.14, 0.18); *n* = 669 (75.6%); <0.0001	+0.15 (0.13, 0.16); *n* = 704 (80.1%); <0.0001	+0.14 (0.13, 0.16); *n* = 727 (82.4%); <0.0001
EQ‐5D VAS	+3.6 (1.6, 5.5); *n* = 670 (75.7%); 0.00028	+3.7 (1.8, 5.5); *n* = 670 (75.7%); <0.0001	+3.9 (1.9, 5.8); *n* = 705 (80.2%); <0.0001	+3.4 (1.7, 5.0); *n* = 729 (82.7%); <0.0001
% Achieving MCID iHOT‐12	65.6 (429 of 654)	67.1 (440 of 656)	69.0 (470 of 681)	67.9 (478 of 704)
% Achieving SCB iHOT‐12	44.2 (289 of 654)	46.0 (302 of 656)	49.6 (338 of 681)	46.2 (325 of 704)
Mean change at 12‐month, (95%CI); *n* (%); *p* ^a^				
iHOT‐12	+26.0 (23.8, 28.2); *n* = 625 (70.6%); <0.0001	+24.8 (22.6, 27.0); *n* = 632 (71.4%); <0.0001	+25.6 (23.4, 27.9); *n* = 626 (71.2%); <0.0001	+27.4 (25.4, 29.4); *n* = 637 (72.2%); <0.0001
EQ‐5D index	+0.15 (0.13, 0.17); *n* = 654 (73.9%); <0.0001	+0.15 (0.13, 0.17); *n* = 648 (73.2%); <0.0001	+0.13 (0.12, 0.15); *n* = 646 (73.5%); <0.0001	+0.15 (0.13, 0.17); *n* = 661 (74.9%); <0.0001
EQ‐5D VAS	+3.9 (1.9, 5.8); *n* = 664 (75.0%); 0.00011	+2.1 (0.1, 4.1); *n* = 649 (73.3%); 0.041	+5.0 (3.1, 7.0); *n* = 646 (73.5%); <0.0001	+4.4 (2.7, 6.2); *n* = 661 (74.9%); <0.0001
% Achieving MCID iHOT‐12	64.8 (405 of 625)	63.4 (401 of 632)	64.2 (402 of 626)	71.1 (453 of 637)
% Achieving SCB iHOT‐12	45.8 (286 of 625)	45.6 (288 of 632)	47.0 (294 of 626)	50.9 (324 of 637)

Abbreviations: CI, confidence interval. EQ‐5D, EuroQol‐5 Dimensions (EQ‐5D) Index; iHOT‐12, 12‐question International Hip Outcome Tool; IQR, interquartile range; MCID, minimum clinically important difference; Preop, preoperative; SCB, substantial clinical benefit; SD, standard deviation; VAS, visual analogue scale.

^a^ The values are given as the mean score improvement (95% CI); number (%) of cases available for follow‐up; *p*‐value derived with the paired t test comparing pre versus postoperative score. MCID and SCB are predicated on pre and postoperative scores to be recorded.

The highest annual volume quartile group had a significantly higher proportion of cases achieving MCID compared to the 2nd lowest cumulative volume quartile at 12 months postoperatively (*p* = 0.03).

### Multivariable analysis

Annual consultant surgeon HA volume demonstrated a trend towards higher postoperative iHOT‐12 score gain at 6 and 12 months as annual HA volume increased, however this was not statistically significant (Figure 2). Our findings demonstrated a significant association between higher cumulative HA volume and greater postoperative iHOT‐12 score improvement at 12 months; cases where the consultant had undertaken a cumulative volume of 170 HA cases had the lowest predicted postoperative iHOT‐12 score gain (29.6 [95% CI: 27.0–32.1]) which was significantly lower than cases where the consultant had undertaken a cumulative volume of 972 (35.2 [95% CI: 32.1–38.3]) where the 95% confidence intervals no‐longer overlapped. Multivariate modelling did not reveal any significant associations between annual or cumulative consultant HA volume and postoperative EQ‐5D score improvement at 6 or 12 months.

**Figure 2:**
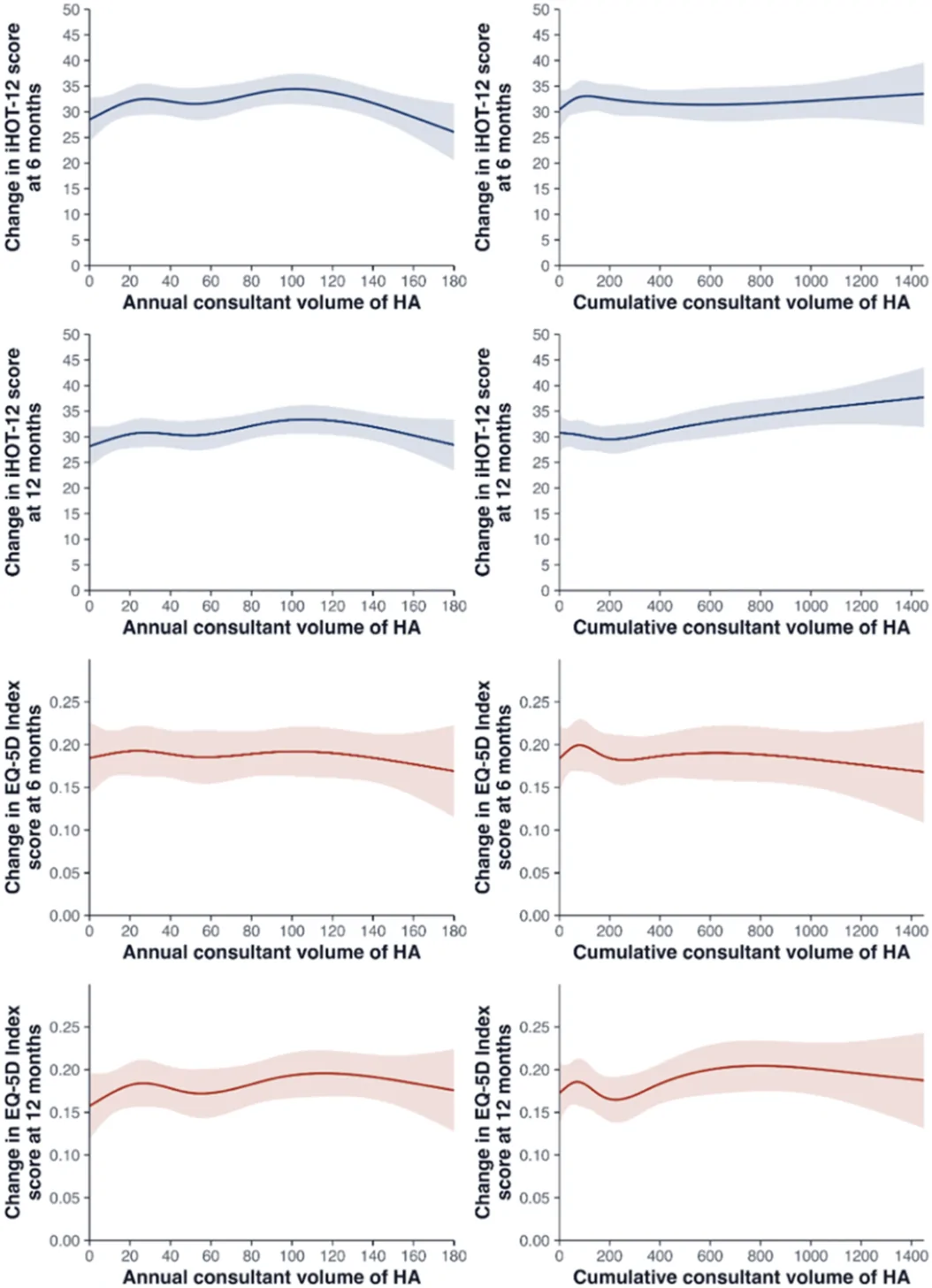
Plot matrix showing the marginal association of change in surgical volume on the postoperative outcome scores following hip arthroscopy after adjustment for confounding factors. Adjustment variables were considered to be at their reference values (female, age 36, cam impingement FAIS subtype, preoperative iHOT‐12 = 31, Grade 1 or 2 acetabular chondral damage). Shaded areas represent 95% confidence interval. EQ‐5D, EuroQol‐5 dimensions (EQ‐5D) index; HA, hip arthroscopy; iHOT‐12, 12‐question International Hip Outcome Tool.

## DISCUSSION

This study observed a statistically significant improvement in postoperative iHOT‐12 score in association with higher cumulative surgeon HA volume. Additionally, we found that younger patients with less‐severe chondral injury grade typically underwent HA by more experienced surgeons. These findings provide important data relating to surgeons and centres providing low‐volume, technically challenging hip surgery and may help inform patient selection criteria.

Preoperative iHOT‐12 scores were highest in the highest‐volume quartile of cumulative surgeon experience. This patient group had the youngest mean age and the least‐significant articular cartilage damage, which likely contributes to their higher preoperative iHOT‐12 scores; it may be that surgeons who perform the highest volumes of HAs practice in specialist centres where HA is considered earlier in the disease course, selecting patients who are younger with less‐severe cartilage damage and therefore higher preoperative iHOT‐12 scores. Shankar et al. reported that higher‐volume hip arthroscopy centres in the USA were found to be operating on younger patients when compared to lower‐volume centres [45], whilst Haipeng et al. found that more experienced HA surgeons performed a greater proportion of isolated cam excisions [14]. Co‐existent OA is associated with worse outcomes from HA [21, 27], and the selection of patients with less severe cartilage damage, and therefore higher preoperative PROM scores, is likely a key driver for the greater postoperative PROM scores described. Statistically‐significantly higher absolute 12‐month postoperative iHOT‐12 scores were observed between the highest cumulative volume surgeon quartile and the lowest two cumulative volume quartiles; however, no difference in mean iHOT‐12 score improvement was noted between any groups. Our findings suggest that more experienced surgeons are more effective at patient selection, leading to narrower selection criteria, interventions on younger patients with less severe chondral damage and therefore better absolute outcomes, a finding which is supported in both orthopaedic and nonorthopaedic literature [2, 12, 14, 15, 51].

The observation that the highest‐volume surgeons treat younger patients with less advanced joint degeneration may also reflect established referral pathways that may preferentially direct younger, more active patients to higher‐volume subspecialist centres. Athletes and younger active individuals, who represent optimal candidates for hip arthroscopy and (almost by definition) will have a lower proportion of degenerative changes in the joint, are often managed through sports medicine networks that refer patients directly to high‐volume surgeons and/or centres with established expertise in treating FAIS. These patients typically present earlier in the disease process, have higher functional demands and possess superior healing capacity compared to older cohorts. The concentration of younger patients in high‐volume practices may therefore represent both selective referral patterns and the natural evolution of subspecialisation, where surgeons developing expertise in HA become the preferred destination for younger patients seeking to return to high‐level activity. This creates a self‐reinforcing cycle where high‐volume surgeons, through treating patients with inherently better prognosis, achieve superior outcomes that further enhance their reputation and referral base. Consequently, despite attempts to adjust our models, the volume‐outcome relationship observed may partially reflect patient characteristics and referral bias rather than purely technical surgical factors. This confounding makes it difficult to determine whether superior outcomes drive referral patterns and higher volumes, or whether the selective treatment of lower‐risk patients by high‐volume surgeons creates an apparent volume‐outcome relationship. Furthermore, the highest‐volume quartile contained only five surgeons, raising concerns about both the generalisability of these findings and the potential for outlier effects to disproportionately influence the observed volume‐outcome relationship.

Multivariable analysis demonstrated a statistically significant increase in postoperative iHOT‐12 score improvement at 12 months postoperatively with increasing surgeon cumulative HA volume. It should be noted that this difference in iHOT‐12 score was small (only 5.6 points—less than the MCID) and that for all modelled values of cumulative surgeon HA volume, there was significant improvement in baseline iHOT‐12 score above the MCID (Figure 2), thus these differences are unlikely to be clinically significant. There was also a trend for increased postoperative iHOT‐12 improvement at 6 and 12 months with increasing surgeon annual volume, but this did not reach statistical significance. These findings suggest that cumulative surgeon volume may be more important than annual volume for achieving favourable postoperative outcomes, a finding which has been supported by other studies in hip replacement and nonorthopaedic surgical specialties [16, 41]. It should be noted, however, that increasing annual case volume has been shown to improve outcomes from primary total hip arthroplasty [49].

Several studies have been published assessing the effects of increasing surgeon HA volume on PROMs postsurgery, with little consensus reached [7, 9, 20, 25, 26, 30, 35, 43, 48]. The lack of clear evidence of a significant improvement in outcomes with increasing surgeon case volume in previous studies combined with the relatively modest improvement in 12‐month iHOT‐12 score improvement described in the present study may indicate a less major relationship between surgical volume and outcome in the context of HA for FAI, which has perhaps not been consistently detected in previous studies that utilise smaller cohorts or include a fewer number of surgeons than the present study. The strength of the present study in case and surgeon numbers may have improved the present study's abilities to detect more subtle change than in the previous literature. There is a larger literature base surrounding the effects of volume on postoperative complications and reoperation rates, with the majority reporting that as surgical volume increases, adverse outcomes decrease, although there is no consensus on which adverse outcomes are decreased most consistently [7, 10, 17, 20, 25, 26, 35, 43, 45, 48, 52, 55]. Notably, some studies have reported no improvement in adverse outcomes with greater surgical experience [8, 9, 14, 36], and one study described an increase in the rate of 2‐year revision THA in the highest‐volume surgeon group [32]. Studies consistently report decreased total operative and traction times as operative volume increases [26, 33, 39]. This heterogeneity in findings may reflect differences in study design, outcome measures, patient populations and the complex relationship between surgeon selection criteria and case complexity that varies across different countries, healthcare systems and practice settings.

The findings from this study suggest that a learning curve for HA does exist, but over a longer timeframe than previous studies suggest. It may take up to 15 years for surgeons to reach a performance plateau [13], and the findings from this study demonstrate that a relatively large number of cases are required before a statistically significant improvement in outcomes is observed. The large case volume required to achieve significantly better results may explain why the pairwise comparisons in this study did not demonstrate as strongly significant results as the multivariable analysis, as the quartiles were too wide to detect significant change. Our findings are supported by other studies reporting that greater cumulative case volume improves postoperative outcomes [51].

An additional threshold for improved outcomes may exist early in a surgeon's experience, but this may not have been detected in the present study, as HA was already in routine use in the UK prior to the establishment of the NAHR. Many surgeons may have surpassed this threshold before recording procedures in the NAHR, which may lead to an overestimation of the case volume required to achieve more favourable outcomes in the present study. Notwithstanding the above limitations, the volume‐outcome charts (Figure 2) reveal noteworthy, but not statistically significant, trends in the early learning phase; the graphs demonstrate notably steep gradients in iHOT‐12 score improvement during the initial 100 cumulative cases, particularly evident at 6 months for both iHOT‐12 and EQ‐5D score change. This pattern supports the notion that the most substantial gains in patient outcomes occur during the early learning curve, consistent with established surgical learning theory, where initial case experience yields the greatest marginal benefit. The steepest portion of the learning curve in the present study appears to plateau around 100–200 cumulative cases, after which further volume increases produce more modest incremental improvements. This finding aligns with previous studies suggesting that fundamental technical competency in HA may be achieved relatively early in a surgeon's experience, but that continued refinement of patient selection and surgical technique occurs over a much longer timeframe.

No significant associations were found between cumulative or annual surgeon volumes and postoperative EQ‐5D scores. EQ‐5D is a general measure of health and our findings suggests that the EQ‐5D is less sensitive for assessing the impact of hip pathology than the iHOT‐12.

The findings of our study have several implications for clinical practice. First, the association between higher cumulative volume and better functional outcomes supports the continued development and maintenance of subspecialist, higher‐volume HA services and, where appropriate, referral of patients to such centres. Second, the observation that higher‐volume surgeons operated on younger patients with less advanced chondral damage, who achieved better absolute outcomes, supports the timely identification and referral of suitable candidates earlier in the disease course. Third, because the volume‐outcome association was attenuated by case‐mix and referral patterns, volume‐based metrics should be interpreted cautiously when applied to service planning or credentialing. The cumulative volumes at which predicted iHOT‐12 improvement differed significantly (approximately 170 vs. 972 cases) should be regarded as a description of where differences became statistically discernible rather than as a prescriptive competency threshold.

### Limitations

As discussed above, the data from this study may overestimate the duration of the learning curve for HA. We acknowledge missing PROMS outcome data at 6 and 12 months. Poor PROM response rates are common in the literature and can range from 57.2% to 68.6% [5, 13, 28, 38, 40, 47]. In the author's experience, patients with the most favourable outcomes are most commonly lost to follow‐up, a finding which is supported in the literature [5]. Other studies have reported no differences in outcomes between attenders and nonattenders [1, 39]. The results of this study may therefore under‐estimate the favourable outcomes of HA, as a greater proportion of the patients successfully followed‐up may have experienced poorer outcomes, leading to potential transfer bias. In contrast, recording a pathway in the NAHR is voluntary, which may lead to omission of patients at a greater risk of experiencing poorer outcomes, which may skew the results towards better outcomes. The observational nature of the present study cannot directly infer case complexity or decision‐making in case selection and so cannot effectively evaluate causality of findings. We were also unable to exclude residual confounding; despite multivariable adjustment, the observed association may partly reflect unmeasured differences in case‐mix and established referral pathways rather than surgeon volume per se. In addition, cases performed by the same surgeon are not (in reality) statistically independent, and we did not employ surgeon‐level random‐effects (mixed‐effects) modelling to account for clustering. This, combined with the small number of surgeons in the highest‐volume quartile (*n* = 5), may limit the robustness and generalisability of the volume‐outcome estimates. Future analyses incorporating the surgeon as a random effect would provide more robust estimates.

Significant intrapersonal variation in PROMs for musculoskeletal conditions, such as FAIS, has been reported in the literature [6]. Additionally, PROMs are subject to response shift and response bias [22]. The use of MCID risks misclassifying those below the cut‐off as ‘not improved’ even if the patient has experienced meaningful benefit [54], a scenario that has been reported in previous studies [44]. The MCID may differ depending on the population, baseline PROM score, sample size and method used to calculate MCID and is prone to cofounding and susceptible to extrinsic factors of the study population [4, 31, 44], and so using a single value may not be applicable to all patients.

## CONCLUSION

This study demonstrates a statistically significant association between cumulative surgeon volume and postoperative improvement in iHOT‐12 scores up to 12 months following hip arthroscopy. The highest‐volume surgeons consistently treated younger patients with less severe chondral damage. The results support the continued development of subspecialist hip arthroscopy services while emphasising the importance of appropriate patient selection for HA. Although statistically significant, the magnitude of the volume‐related improvement in iHOT‐12 score was small and did not reach the minimum clinically important difference. The association may partly reflect case‐mix and referral patterns rather than surgical volume alone. Nonetheless, to our knowledge, this represents the largest national registry cohort to examine this question and the first to model surgeon volume as a continuous exposure, supporting the development of subspecialist HA services and the timely referral of appropriately selected patients.

## AUTHOR CONTRIBUTIONS

**Richard Holleyman**: Conceptualisation; data curation; formal analysis; investigation; methodology; project administration; resources; software; supervision; validation; visualisation; writing—original draft; writing—review and editing. **William Cawley**: Investigation; project administration; writing—original draft; writing—review and editing. **Tim Board**: Writing—original draft; writing—review and editing. **Marcus Bankes**: Writing—review and editing. **Antonio Andrade**: Writing—review and editing. **Andrew Judge**: Methodology; supervision; writing—review and editing. **Vikas Khanduja**: Conceptualisation; methodology; supervision; writing—review and editing. **Bernard van Duren**: Writing—original draft; writing—review and editing. **Ajay Malviya**: Conceptualisation; methodology; project administration; supervision; writing—original draft; writing—review and editing. All authors read and approved the final manuscript.

## ACKNOWLEDGEMENTS

Author Richard Holleyman was supported by a grant from Orthopaedic Research UK (Ref 541) to undertake this study.

## CONFLICT OF INTEREST STATEMENT

Richard Holleyman: Richard Holleyman reports financial support was provided by Orthopaedic Research UK. William Cawley: No known competing financial interests or personal relationships that could have appeared to influence the work reported in this paper Tim Board: Tim N. Board reports a relationship with British Hip Society that includes: serving on Executive Board. Marcus Bankes: No known competing financial interests or personal relationships that could have appeared to influence the work reported in this paper. Antonio Andrade: Antonio J. Andrade reports a relationship with Stryker Orthopaedics that includes: speaking and lecture fees. Antonio J. Andrade reports a relationship with Smith & Nephew Inc that includes: consulting or advisory, funding grants and speaking and lecture fees. Antonio J. Andrade reports a relationship with Journal of Hip Preservation Surgery that includes: board membership. Antonio J. Andrade reports a relationship with International Society for Hip Arthroscopy that includes: board membership. Antonio J. Andrade reports a relationship with British Hip Society that includes: board membership. Andy Judge: Andrew Judge reports financial support was provided by University of Bristol. Andrew Judge reports a relationship with University of Bristol that includes: funding grants. AJ has received research funding to their institution from the NIHR, HDR UK, versus Arthritis, Healthcare Quality Improvement Partnership, Royal College of Physicians, Tommy's and Health Foundation. Andy Judge was partially supported by the National Institute for Health and Care Research (NIHR) Bristol Biomedical Research Centre and the NIHR Applied Research Collaboration (ARC) West. Vikas Khanduja: Vikas Khanduja reports a relationship with Stryker Orthopaedics, Smith and Nephew, Arthrex, SICOT, ISHA, JBJS (Am), BOA and NAHR that includes: board membership, consulting or advisory and speaking and lecture fees. Bernard van Duren: No known competing financial interests or personal relationships that could have appeared to influence the work reported in this paper. Ajay Malviya: Ajay Malviya reports a relationship with Smith & Nephew Inc that includes: consulting or advisory.

## ETHICS STATEMENT

Please include the name of the institutional review board (IRB) and the approval number. If not applicable, please state so. NAHR research and scientific committee (reference: NAHR/2023/09).

## Data Availability

The data that support the findings of this study are available on request from the corresponding author. The data are not publicly available due to privacy or ethical restrictions.
